# Cascade biotransformation of dehydroepiandrosterone (DHEA) by *Beauveria* species

**DOI:** 10.1038/s41598-018-31665-2

**Published:** 2018-09-07

**Authors:** Ewa Kozłowska, Monika Urbaniak, Natalia Hoc, Jakub Grzeszczuk, Monika Dymarska, Łukasz Stępień, Elżbieta Pląskowska, Edyta Kostrzewa-Susłow, Tomasz Janeczko

**Affiliations:** 1 Department of Chemistry, Wrocław University of Environmental and Life Sciences, Norwida 25, 50-375 Wrocław, Poland; 2 Department of Pathogen Genetics and Plant Resistance, Institute of Plant Genetics, Polish Academy of Sciences, Strzeszyńska 34, 60-479 Poznań, Poland; 3 Department of Plant Protection, Division of Phytopathology and Mycology, Wrocław University of Environmental and Life Sciences, pl. Grunwaldzki 24a, 50-363 Wrocław, Poland

## Abstract

*Beauveria bassiana* is an entomopathogenic fungus used as a biological control agent. It is a well-known biocatalyst for the transformation of steroid compounds. Hydroxylations at the 7α or 11α position and oxidation to D-homo lactones are described in the literature. In our study, we examined the diversity of metabolism of five different *B*. *bassiana* strains and compared them to already known pathways. According to the literature, 7α and 11α-hydroxy derivatives as well as 3β,11α-dihydroxy-17a-oxa-D-homo-androst-5-en-17-one have been observed. Here we describe new DHEA metabolic pathways and two products not described before: 3β-hydroxy-17a-oxa-D-homo-androst-5-en-7,17-dione and 3β,11α-dihydroxyandrost-5-en-7,17-dione. We also used for the first time another species from this genus, *Beauveria caledonica*, for steroid transformation. DHEA was hydroxylated at the 7α, 7β and 11α positions and then reactions of oxidation and reduction leading to 3β,11α-dihydroxyandrost-5-en-7,17-dione were observed. All tested strains from the *Beauveria* genus effectively transformed the steroid substrate using several different enzymes, resulting in cascade transformation.

## Introduction

Among steroid hormones, dehydroepiandrosterone (DHEA) is the most abundant adrenal hormone circulating in human blood. DHEA is synthesised from cholesterol mainly in adrenal glands but also in testes, ovaries and brain^1^. It is metabolised by bone, muscle, breast, skin and adipose tissue as well as brain, intestine and liver^1,2^. DHEA is transformed further, to biologically active androgens and estrogens, but its hydroxy derivatives play a significant role^2,3^. Oxygenated derivatives can affect memory and cognitive functions^4–6^, rheumatologic arthritis^7,8^, colitis^9–11^, thermogenesis^12,13^, the immune response and autoimmune diseases^14–17^. At this stage of knowledge, the most promising as drugs are 7α- and 7β-hydroxy-DHEA, the main products of oxidation. They are formed from DHEA in liver, skin and brain by CYP7B1 (7α-hydroxylase) and by interconversion via 7-oxo-DHEA to 7β-hydroxy-DHEA by 11β-hydroxysteroid dehydrogenase type I (11β-HSD1)^2,18^. The level of DHEA in the cerebrospinal fluid is higher than the blood level and it has neuroprotective activities. Lowered expression of CYP7B1 in the human brain was observed in Alzheimer disease^2,19^. As mentioned above, oxygenated derivatives of DHEA may play an important role in many functions and diseases.

Biotransformation of steroid compounds is an easy method of obtaining hydroxy derivatives at inactivated positions. Although microbial strains capable of hydroxylating at any steroid position (except C4, 10 and 13) are known, there is still a need to screen for a new organism than can perform the desired transformations^20^. Selecting species for metabolism testing should not be entirely based on literature data. *Fusarium acuminatum* KCh S1 and *Mucor hiemalis* KCh W2 provide hydroxylation only at the 7α position, similar to *Aspergillus versicolor* KCh TJ1, while *Penicillium commune* KCh W7 and *Penicillium chrysogenum* KCh S4 do not hydroxylate DHEA at C7, in contrast to literature data^21,22^. However, every strain should be treated individually. All 13 strains of *Isaria farinosa* used in our previous studies provide the same reaction for DHEA, while this study presents five different transformation pathways for *Beauveria bassiana* strains^23^.

Entomopathogenic fungi, such as *Isaria* and *Beauveria*, are natural regulatory factors in insect populations. Among them, only *Beauveria*, *Isaria* and *Metarhizium* are used as biocatalysts for the transformation of various compounds^24–29^.

*Beauveria caledonica* strains excrete metabolites which provide a source of protons and metal-chelating properties, e.g., carboxylic acids, amino acids, siderophores and phenolic compounds, which take part in dissolution and formation of minerals^30^ as well as in transformation and bioremediation of toxic metals (cadmium, copper, lead, zinc, uranium)^31–33^. *B*. *caledonica* is also used in sulfoxidation of amino acids^34,35^ but has never been used in the transformation of steroid compounds so far.

*Beauveria bassiana* is one of the most ubiquitous and extensively studied of the entomopathogenic fungal species, both as a biological control agent and as a biocatalyst. This species is known for its ability to transform a variety of substrates such as aromatic amines^36^, cyclic and aliphatic ketones^37,38^, terpenes and terpenoids^39,40^, flavonoids^41–43^, amino acids^34,35^ and steroids^22,24,44–50^. This biocatalyst’s enzymes provide reactions including hydroxylation^22,24,46–48,51–53^, sulfoxidation^34,35^, dealkylation^25^, glucosidation^36,39,42,43,54^ or deglycosylation^55^, acetylation^25,36^, epoxidation^56^, Baeyer–Villiger oxidation^44,46^, reduction and ester hydrolysis^25^. It seems that the popularity of *Beauveria bassiana* as a catalyst has been increasing in recent years. These species provide mainly hydroxylation at the 11α position of the steroid skeleton^24,25,44–47,51^, less frequently oxidation of the D ring in Baeyer-Villiger type^44,46^. Predominantly, hydroxylation of DHEA by these species is proceeded by reduction of the carbonyl group at C-17^46,51,57^. In several cases, this hydroxyl group (C-17) is oxidised, which is the first step in the formation to the corresponding D-lactone^46^. Moreover, papers which describe the biotransformations of DHEA by strains of this genus present various metabolites. For this reason, new strains of *Beauveria bassiana* and *Beauveria caledonica* isolated from insects‘ cadavers founded in abandoned mines were investigated for their capacity to transformation of dehydroepiandrosterone.

## Materials and Methods

### Materials

The substrate, dehydroepiandrosterone (DHEA, 3β-hydroxyandrost-5-en-17-one) (**1**), was purchased from Sigma-Aldrich.

#### Microorganisms

The two strains of *Beauveria caledonica* (KCh J3.3 and KCh J3.4) and five strains of *Beauveria bassiana* (KCh J1.5, KCh J2.5, KCh BBT, KCh J1, KCh J3.2) were maintained on Sabouraud 4% dextrose-agar slopes and freshly subcultured before use.

### Isolation of strains

The studied fungi (except *Beauveria bassiana* KCh BBT) were collected in abandoned mines located near Ciechanowice, Lower Silesian Voivodeship, Poland in winter 2014/2015. *Beauveria bassiana* KCh BBT was collected in Tenerife. Found cadavers of insects overgrown by the fungal hyphae were placed in sterile plastic containers. The samples were transported to the laboratory of the Plant Pathology and Mycology Division of the Department of Plant Protection, Wrocław University of Environmental and Life Sciences. There, pieces of hyphae were placed on Petri dishes with cultivation medium. To obtain high-quality material for genomic DNA extraction, fungal strains were cultivated for 7 days on potato dextrose agar medium (PDA, Oxoid).

### DNA extraction and molecular identification of fungal strains

A modified method using CTAB (hexadecyltrimethylammonium bromide) was applied for genomic DNA extraction, as described earlier^58^. Species identification was performed on the basis of the sequence analysis of the Internal Transcribed Spacers of the ribosomal DNA region (ITS1-ITS2).

Polymerase chain reactions (PCRs) were performed as described earlier^27^ using DreamTaq Green DNA polymerase (Thermo Scientific, Espoo, Finland). For the PCR amplification specific primers were used: ITS4 – forward primer (5′-TCCTCCGCTTATTGATATGC-3′) and ITS5 – reverse primer (5′-GGAAGTAAAAGTCGTAACAAGG-3′)^59^. Amplicons were separated in 1.5% agarose gel (Invitrogen) with GelGreen Nucleic Acid Stain (Biotium, Inc.).

For sequence analysis, PCR-amplified DNA fragments were purified as described earlier^60^. DNA fragments were labelled using a forward primer and the BigDyeTerminator 3.1 kit (Applied Biosystems, Foster City, CA, USA), according to the producer’s recommendations and precipitated with 96% ethanol. Sequence reading was performed using Applied Biosystems equipment. Sequences were analysed using the BLASTn algorithm against the GenBank database-deposited reference sequences.

### Steroid biotransformation

One hundred millilitres of the cultivation medium (3 g of glucose and 1 g of aminobac dissolved in water) in Erlenmeyer flasks (300 ml) was inoculated with a suspension of microorganisms and then incubated for 3 days at 25 °C on a rotary shaker. Then 10 mg of DHEA (**1**) dissolved in 1 ml of THF was added. After 3, 6, 9 and 12 hours and at 1, 3, 7 and 10 days of incubation under the above conditions, portions of 10 ml of the transformation mixture were taken out and extracted with chloroform. The extracts were dried over MgSO_4_, concentrated *in vacuo* and analysed by gas chromatography (GC) and thin-layer chromatography (TLC). All the experiments were repeated three times.

### Preparative biotransformation

Selected transformations were performed on the preparative scale in 2000 ml flasks, each containing 500 ml of the cultivation medium. After 3-day incubation (conditions as above) 100 mg of substrate dissolved in 2 ml of THF was added. After the time specified for each transformation, the medium was extracted with CHCl_3_ (3 × 300 ml), dried (MgSO_4_) and concentrated *in vacuo*. The transformation products were separated by preparative TLC and analysed (TLC, GC, NMR).

### Analytical methods

The course of biotransformation was monitored using TLC. The composition of product mixtures was established by GC. Products were separated using preparative TLC plates (Silica Gel GF, 20 × 20 cm, 500 μm, Analtech) and a hexane/acetone mixture (2:1, v/v) as an eluent. Analytical TLC was carried out on silica gel G (Merck). Compounds were detected by spraying the plates with a H_2_SO_4_/CH_3_OH mixture (1:1, v/v). GC analysis was performed using a Hewlett-Packard 5890 A (Series II) GC instrument fitted with a flame ionisation detector (FID). A HP-5 (crosslinked phenyl methyl siloxane) capillary column (30 m × 0.32 mm × 0.25 μm) was used to determine the composition of product mixtures. The following temperature programme was used: 220 °C (1 min)/4 °C/min/260 °C (1 min)/30 °C/min/300 °C (5 min). The NMR spectra were recorded on a DRX 500 MHz Bruker spectrometer and measured in CDCl_3_.

## Results and Discussion

### Spectral data and isolated yields of products

#### 3β,7α-dihydroxyandrost-5-en-17-one (**2**)

28.5 mg of **2** was isolated after 24-hour transformation of **1** (100 mg) in the *Beauveria caledonica* KCh J3.4 culture; after 12-hour transformation of 100 mg of **1** in the *B*. *bassiana* KCh J1.5 culture 23.7 mg of **2** was isolated.

^1^H NMR (600 MHz) (ppm) (CDCl_3_) δ: 0.87 (s, 3H, 18-H); 1.01 (s, 3H, 19-H); 1.11 (td, 1H, *J* = 13.4, 3.8 Hz, 1-Hα); 1.23–1.31 (m, 2H, 9-H, 12-Hα); 1.49 (td, 1H, *J* = 13.1, 4.3 Hz, 11-Hα); 1.50–1.60 (m, 2H, 2-Hα, 15-Hα); 1.64–1.72 (m, 2H, 8-H, 11-Hβ); 1.78 (td, 1H, *J* = 12.1, 5.3 Hz, 14-H); 1.80–1.89 (m, 3H, 1-Hβ, 2-Hβ, 12-Hβ); 2.07–2.17 (m, 2H, 15-Hβ, 16-Hα); 2.29 (br t, 1H, *J* = 12.3 Hz, 4-Ha); 2.35 (ddd, 1H, *J* = 13.3, 4.8, 2.0 Hz, 4-Hβ); 2.33 (dd, 1H, *J* = 13.1, 4.6 Hz, 16-Hβ); 3.56 (tt, 1H, *J* = 11.3, 4.7 Hz, 3-Hα); 3.96 (br t, 1H, *J* = 3.8 Hz, 7-Hβ) 5.63 (dd, 1H, *J* = 5.1, 1.2 Hz, 6-H).

#### 3β,11α-dihydroxyandrost-5-en-17-one (**3**)

After 12-hour transformation of 100 mg of **1** in the *B. bassiana* KCh J1.5 culture the isolation yield was 5.5 mg of **3**.

^1^H NMR (600 MHz) (ppm) (CDCl_3_) δ: 0.90 (s, 3H, 18-H); 1.07 (t, 1H, *J* = 10.3 Hz, 9-H); 1.18 (td, 1H, *J* = 14.0, 3.8 Hz, 1-Hα); 1.19 (s, 3H, 19-H); 1.31–1.42 (m, 2H, 14-H, 12-Hα); 1.50–1.64 (m, 3H, 2-Hα, 8-H, 15-Hα); 1.65–1.72 (m, 1H, 7-Hα); 1.81 (dm, 1H, *J* = 12.8 Hz, 2-Hβ); 1.97 (dddd, 1H, *J* = 15.6, 7.9, 4.7, 0.8 Hz, 15-Hβ); 2.09 (ddd, 1H, *J* = 13.0, 5.6, 2.3 Hz, 7-Hβ);2.14 (dt, 1H, *J* = 19.7, 9.2 Hz, 16-Hα); 2.16 (dq, 1H, *J* = 13.4, 3.2 Hz, 4-Hα); 2.28 (tq, 1H, *J* = 10.7, 2.3 Hz, 12-Hβ); 2.33 (ddd, 1H, *J* = 12.9, 5.1, 2.4 Hz, 4-Hβ); 2.49 (dd, 1H, *J* = 19.9, 8.5 Hz, 16-Hβ); 2.58 (dt, 1H, *J* = 13.7, 3.6 Hz, 1-Hβ); 3.54 (tt, 1H, *J* = 11.0, 4.9 Hz, 3-Hα); 4.12 (td, 1H, *J* = 10.6, 4.9 Hz, 11-Hβ); 5.44 (dt, 1H, *J* = 5.7, 1.6 Hz, 6-H).

#### 3β,7β-dihydroxyandrost-5-en-17-one (**4**)

19.2 mg of **4** was isolated after 24-hour transformation of **1** (100 mg) in the *B*. *caledonica* KCh J3.4 culture.

^1^H NMR (600 MHz) (ppm) (CDCl_3_) δ: 0.88 (s, 3H, 18-H); 1.02–1.07 (m, 1H, 1-Hα); 1.06 (s, 3H, 19-H); 1.09 (td, 1H, *J* = 11.9, 4.4 Hz, 9-H); 1.23 (td, 1H, *J* = 13.2, 4.2 Hz, 12-Hα); 1.42 (td, 1H, *J* = 11.9, 5.6 Hz, 14-H); 1.44–1.62 (m, 3H, 2-Hα, 8-H, 11-Hα); 1.65–1.70 (m, 1H, 11-Hβ); 1.80–1.89 (m, 4H, 1-Hβ, 2-Hβ, 12-Hβ, 15-Hα); 2.09 (ddd, 1H, *J* = 19.2, 9.5 Hz, 16-Hα); 2.20–2.28 (m, 2H, 4-Ha, 15-Hβ); 2.33 (dd, 1H, *J* = 12.9, 4.6, 1.6 Hz, 4-Hβ); 2.45 (dd, 1H, *J* = 19.3, 8.8 Hz, 16-Hβ); 3.53 (tt, 1H, *J* = 11.0, 4.7 Hz, 3-Hα); 3.93 (br d, 1H, *J* = 8.3 Hz, 7-Hα); 5.30 (br s, 1H, 6-H).

#### 3β-hydroxyandrost-5-en-7,17-dione (**5**)

Twelve-hour transformation of 100 mg of **1** in the *Beauveria bassiana* KCh BBT culture yielded 7.4 mg of **5**; 9.7 mg of **5** was isolated after 24-hour transformation of **1** (100 mg) in the *B*. *caledonica* KCh J3.4 culture.

^1^H NMR (600 MHz) (ppm) (CDCl_3_) δ: 0.89 (s, 3H, 18-H); 1.22 (s, 3H, 19-H); 1.23–1.34 (m, 2H, 1-Hα, 12-Hα); 1.54–1.68 (m, 4H, 2-Hα, 9-H, 11-Hα, 14-H); 1.70–1.91 (m, 3H, 11-Hβ, 12-Hβ, 15-Hα); 1.91–2.00 (m, 2H, 1-Hβ, 2-Hβ,); 2.14 (dd, 1H, *J* = 18.8, 9.8 Hz, 16-Hα); 2.35–2.45 (m, 2H, 4-Ha, 8-H); 2.46 (dd, 1H, *J* = 18.8, 8.3 Hz, 16-Hβ); 2.54 (ddd, 1H, *J* = 13.9, 4.6, 2,2 Hz, 4-Hβ); 2.81 (ddd, 1H, *J* = 13.4, 8.7, 7.2 Hz, 15-Hβ); 3.68 (tt, 1H, *J* = 11.3, 4.8 Hz, 3-Hα); 5.74 (br s, 1H, 6-H).

#### 3β,11α-dihydroxyandrost-5-en-7,17-dione (**6**)

Twelve-day transformation of 100 mg of **1** in the *Beauveria caledonica* KCh J3.4 culture yielded 14.4 mg of **6**; 5.2 mg of **6** was isolated after 24-hour transformation of **1** (100 mg) in the *B*. *caledonica* KCh J3.4 culture; after 8-day transformation of **1** (100 mg) in the *B*. *bassiana* KCh J1 culture 6.3 mg of **6** was isolated; 12-hour transformation of **1** (100 mg) in the *B*. *bassiana* KCh BBT culture yielded 3.1 mg of **6**.

^1^H NMR (600 MHz) (ppm) (CDCl_3_) δ: 0.90 (s, 3H, 18-H); 1.19–1.31 (m, 2H, 1-Hα, 12-Hα); 1.36 (s, 3H, 19-H); 1.58 (dd, 1H, *J* = 12.9, 9.7 Hz, 9-H); 1.61–1.69 (m, 2H, 2-Hα, 15-Hα); 1.71 (ddd, 1H, *J* = 12.2, 11.0, 5.4 Hz, 14-H); 1.91 (dm, 1H, *J* = 12.4 Hz, 2-Hβ); 2.14 (d, 1H, *J* = 12.4, 5.0 Hz, 12-Hβ); 2.20 (dt, 1H, *J* = 19.8, 9.3 Hz, 16-Hα); 2.41–2.46 (m, 1H, 4-Ha, 8-H); 2.49 (ddd, 1H, *J* = 19.8, 8.9, 0.7 Hz, 16-Hβ); 2.54 (ddd, 1H, *J* = 13.5, 4.9, 2.5 Hz, 4-Hβ); 2.74 (dt, 1H, *J* = 14.3, 3.5 Hz, 1-Hβ); 2.82 (dddd, 1H, *J* = 13.3, 8.9, 5.5, 0.8 Hz, 15-Hβ); 3.68 (t, 1H, *J* = 11.3, 4.8 Hz, 3-Hα); 4.17 (td, 1H, *J* = 10.6, 5.1 Hz, 11-Hβ); 5.78 (d, 1H, *J* = 1.2 Hz, 6-H).

#### 3β,7α,11α-trihydroxyandrost-5-en-17-one (**7**)

After eight-day transformation of 100 mg of **1** in the *Beauveria bassiana* KCh J1 culture 5.1 mg of **7** was isolated.

^1^H NMR (600 MHz) (ppm) (CDCl_3_) δ: 0.90 (s, 3H, 18-H); 1.17 (s, 3H, 19-H); 1.19 (td, 1H *J* = 14.0, 3.7 Hz, 1-Hα); 1.30 (t, 1H, *J* = 11.6 Hz, 12-Hα); 1.37 (dd, 1H, *J* = 11.4, 10.1 Hz, 9-H); 1.54–1.63 (m, 3H, 2-Hα, 8-H, 15-Hα); 1.82–1.88 (m, 2H, 2-Hβ, 14-H); 2.10 (ddd, 1H, *J* = 13.2, 9.4, 5.1 Hz, 15-Hβ); 2.15 (dd, 1H, *J* = 12.2, 5.2 Hz, 12-Hβ); 2.20 (dt, 1H, *J* = 19.3, 9.1 Hz, 16-Hα); 2.32 (ddt, 1H, *J* = 12.8, 11.2, 1.5 Hz, 4-Hα); 2.38 (ddd, 1H, *J* = 13.1, 5.3, 2.3 Hz, 4-Hβ); 2.50 (dd, 1H, *J* = 19.3, 8.6 Hz, 16-Hβ); 2.63 (dt, 1H, *J* = 14.0, 3.6 Hz, 1-Hβ); 3.59 (tt, 1H, *J* = 11.1, 4.9 Hz, 3-Hα); 3.97 (ddd, 1H, *J* = 5.0, 3.8, 1.3 Hz, 7-Hβ); 4.12 (td, 1H, *J* = 13.9, 6.8 Hz, 11-Hβ); 5.70 (dd, 1H, *J* = 5.6, 1.5 Hz, 6-H).

#### 3β-hydroxy-17a-oxa-D-homo-androst-5-en-7,17-dione (**8**)

After 12-hour transformation of 100 mg of **1** in the *Beauveria bassiana* KCh BBT culture 9.6 mg of **8** was isolated.

^1^H NMR (600 MHz) (ppm) (CDCl_3_) δ: 1.20 (s, 3H, 19-H); 1.24 (td, 1H, *J* = 13.4, 3.8 Hz, 1-Hα); 1.32 (s, 3H, 18-H); 1.42–1.54 (m, 2H, 11-Hβ, 15-Hα); 1.59–1.71 (m, 3H, 2-Hα, 9-H, 12-Hα); 1.83 (ddd, 1H, *J* = 13.7, 7.0, 3.9 Hz, 11-Hα); 1.90 (ddd, 1H, *J* = 12.2 11.1, 4.1 Hz, 14-H); 1.93–1.99 (m, 3H, 1-Hβ, 2-Hβ, 12-Hβ); 2.17 (qd, 1H, *J* = 12.0, 10.8 Hz, 8-H); 2.40 (ddd, 1H, *J* = 13.4, 12.1, 1.3 Hz, 4-Ha); 2.54 (ddd, 1H, *J* = 13.9, 4.6, 1.8 Hz, 4-Hβ); 2.66 (ddd, 1H, *J* = 19.0, 8.5, 2.1 Hz, 16-Hβ); 2.72 (dt, 1H, *J* = 18.2, 9.0 Hz, 16-Hα); 2.81 (dddd, 1H, *J* = 13.2, 8.7, 3.6, 2.4 Hz, 15-Hβ); 3.68 (tt, 1H, *J* = 11.0, 4.3 Hz, 3-Hα); 5.74 (br s, 1H, 6-H).

#### 3β,11α-dihydroxy-17a-oxa-D-homo-androst-5-en-17-one (**9**)

Twelve-hour transformation of **1** (100 mg) in the *Beauveria bassiana* KCh BBT culture yielded 4 mg of **9**.

^1^H NMR (600 MHz) (ppm) (CDCl_3_) δ: 1.14 (s, 3H, 19-H); 1.17 (td, 1H, *J* = 10.6, 4.0 Hz, 9-H); 1.27 (qd, 1H, *J* = 12.9, 2.7 Hz, 8-H); 1.32 (s, 3H, 18-H); 1.44–1.59 (m, 3H, 2-Hα, 14-H, 15-Hα); 1.66 (ddd, 1H, *J* = 13.1, 7.4, 2.5 Hz, 1-Hα); 1.75–1.84 (m, 2H, 2-Hβ, 12-Hα); 1.97 (td, 1H, *J* = 9.5, 2.4 Hz, 15-Hβ); 2.15 (dt, 1H, *J* = 14.3, 3.5 Hz, 1-Hβ); 2.24 (dd, 1H, *J* = 11.8, 4.5 Hz, 12-Hβ); 2.33 (ddd, 1H, *J* = 12.9, 5.0, 2.4 Hz, 4-Ha); 2.44 (dd, 1H, *J* = 12.9, 11.1 Hz, 4-Hβ); 2.58 (dd, 1H, *J* = 19.0, 8.4 Hz, 16-Hα); 2.69 (ddd, 1H, *J* = 19.2, 8.2, 2.0 Hz, 16-Hβ); 3.53 (tt, 1H, *J* = 11.2, 4.9 Hz, 3-Hα); 3.88 (ddd, *J* = 11.6, 10.1, 4.6 Hz, 1H); 5.40 (br d, 1H, *J* = 5.7 Hz, 6-H).

#### 3β-hydroxyandrostan-7,17-dione (**10**)

Twelve-day transformation of **1** (100 mg) in the *Beauveria caledonica* KCh J3.4 culture yielded 3 mg of **10**.

^1^H NMR (600 MHz) (ppm) (CDCl_3_) δ: 0.86 (s, 3H, 18-H); 1.22 (s, 3H, 19-H); 1.19–1.34 (m, 4H, 1-Hα, 2-Hβ, 4-Ha, 12-Hα); 1.46–1.54 (m, 2H, 11-Hα, 15-Hα); 1.59–1.67 (m, 1H, 11-Hβ); 1.69–1.77 (m, 3H, 4-Hβ, 9-H, 12-Hβ); 1.82 (ddd, 1H, *J* = 12.5, 4.0, 2.8 Hz, 2-Hα); 1.83–1.89 (m, 2H, 1-Hβ, 5-H); 1.91 (dddd, 1H, *J* = 13.4, 6.1, 3.9, 2.3 Hz, 14-H); 2.01 (dd, 1H, *J* = 10.6, 2.4 Hz, 6-Hα); 2.15 (dt, 1H, *J* = 19.4, 9.2 Hz, 16-Hα); 2.43 (ddd, 1H, *J* = 19.4, 8.3, 1.3 Hz, 16-Hβ); 2.54 (t, 1H, *J* = 11.4 Hz, 8-H); 2.53–2.60 (m, 1H, 15-Hβ); 2.89 (dd, 1H, *J* = 11.9, 6.1, 0.7 Hz, 6-Hβ); 3.62 (t, 1H, *J* = 11.3, 4.8 Hz, 3-Hα).

#### 11α-hydroxyandrostan-3,7,17-trione (**11**)

Twelve-day transformation of **1** (100 mg) in the *Beauveria caledonica* KCh J3.4 culture yielded 2 mg of **11**.

^1^H NMR (600 MHz) (ppm) (CDCl_3_) δ: 0.91 (s, 3H, 18-H); 1.33–1.39 (m, 1H, 12-Hα); 1.45 (s, 3H, 19-H); 1.43–1.49 (m, 1H, 15-Hα); 1.58 (td, 1H, *J* = 13.9, 4.0 Hz, 1-Hα); 1.85–1.89 (m, 2H, 4-Ha, 5-H); 2.02–2.05 (m, 2H, 16-Hα, 9-H); 2.12–2.21 (m, 5H, 1-Hβ, 4-Hβ, 12-Hβ, 14-H, 16-Hα); 2.49 (ddd, 1H, *J* = 19.5, 8.9, 1.1 Hz, 16-Hβ); 2.53–2.60 (m, 1H, 15-Hβ); 2.59–2.65 (m, 1H, 2-Hα); 2.66 (t, 1H, *J* = 11.4 Hz, 8-H); 2.79 (ddd, 1H, *J* = 13.9, 5.3, 3.4 Hz, 2-Hβ); 2.91 (d, 1H, *J* = 11.9, 5.7 Hz, 6-Hβ); 4.22 (td, 1H *J* = 10.6, 5.1 Hz, 11-Hβ).

### Species identification based on the analysis of the ITS region

In this study, fungal strains were identified using sequencing of the PCR-amplified specific genomic region. The DNA fragments were amplified with ITS4 and ITS5 primers, sequenced and compared with reference ITS sequences deposited in the GenBank database (Table 1). The complete sequences of those products indicated over 99% identity to individual ITS sequences. Strains KCh J1, KCh J1.5, KCh J2.5, KCh J3.2 and KCh BBT were identified as the species *Beauveria bassiana*, whereas strains KCh J3.3 and KCh J3.4 were identified as the species *Beauveria caledonica*.

**Table 1:** Fungal strains’ identification based on ITS-ITS2 sequence analysis and their comparison to the reference sequences deposited in the GenBank database.

Fungal strain	Identified species	Sequence nucleotide identity
KCh J1	*Beauveria bassiana*	99% identity to *Beauveria bassiana*, acc. numbers: KX664457.1, KT378231.1, KU198598.1
KCh J1.5	*Beauveria bassiana*	99% identity to *Beauveria bassiana*, acc. numbers: KC753398.1, KC753394.1, KC753392.1
KCh J2.5	*Beauveria bassiana*	99% identity to *Beauveria bassiana*, acc. numbers: KC753394.1, KC753392.1, JX149538.1
KCh J3.2	*Beauveria bassiana*	99% identity to *Beauveria bassiana*, acc. numbers: KY852373.1, KX664457.1, KT378231.1
KCh J3.3	*Beauveria caledonica*	99% identity to *Beauveria caledonica*, acc. numbers: DQ350137.1, NR_077147.1, KY471655.1
KCh J3.4	*Beauveria caledonica*	99% identity to *Beauveria caledonica*, acc. numbers: DQ350137.1, KY471655.1, HQ880816.1
KCh BBT	*Beauveria bassiana*	99% identity to *Beauveria bassiana*, acc. numbers: JQ266190.1, JQ266187.1, DQ449648.1

### Structural identification of products

Metabolism of DHEA (**1**) by tested strains yielded known metabolites identified as 7βOH-DHEA (**4**) and 7αOH-DHEA (**2**) by comparison of their spectral data with the literature values^27^ and on the basis of the identity of their R_t_ from GC and R_f_ from TLC with standards available in our laboratory. The products’ structures were determined by means of ^1^H NMR, ^13^C NMR and correlation spectroscopy. ^13^C NMR spectra of all the products obtained are summarised in Table 2.

**Table 2:** ^13^C NMR chemical shifts of products in CDCl_3_.

Atom number	Products									
	(2)	(3)	(4)	(5)	(6)	(7)	(8)	(9)	(10)	(11)
1	37.07	39.13	37.02	36.60	38.82	38.86	36.28	31.17	31.03	38.09
2	31.39	31.89	31.60	31.25	31.43	31.64	32.07	31.77	34.29	38.19
3	71.25	71.86	71.30	70.44	70.77	71.46	70.55	71.70	70.89	210.89
4	42.06	42.84	41.77	41.99	42.55	42.58	41.68	42.52	37.50	43.36
5	146.64	141.68	143.78	166.27	166.02	147.32	164.98	141.33	43.21	43.94
6	123.67	120.88	125.65	126.06	125.41	123.38	126.99	120.36	45.39	44.95
7	64.37	29.85	72.91	201.20	200.44	64.11	199.93	39.19	211.13	209.66
8	37.32	31.52	40.54	44.47	43.77	36.91	46.49	34.33	48.71	49.25
9	42.72	57.11	48.37	50.22	55.85	49.65	49.11	55.91	44.75	48.42
10	37.63	38.47	36.78	38.54	40.49	39.19	38.93	38.41	35.41	36.74
11	20.19	68.86	20.51	20.72	68.37	68.86	21.79	68.63	21.19	68.24
12	31.18	42.77	31.36	30.85	42.07	42.48	38.08	49.50	29.99	42.31
13	47.23	48.13	47.89	48.00	48.11	47.69	82.43	81.71	47.61	47.69
14	45.05	50.84	51.34	45.88	44.71	44.79	40.46	45.96	45.91	47.50
15	22.02	21.93	24.31	24.31	23.91	21.75	21.42	20.09	23.11	22.97
16	35.91	36.06	36.10	35.77	35.82	36.01	29.08	28.75	35.78	35.80
17	221.30	219.27	221.31	220.52	218.77	219.19	171.73	169.90	220.39	217.32
18	13.39	14.44	13.70	13.89	14.67	14.24	20.98	21.11	13.99	14.83
19	18.38	19.27	19.29	17.57	17.26	18.15	17.03	19.01	22.87	22.63

In comparison to 7αOH-DHEA (**2**) and 7βOH-DHEA (**4**), in the ^1^H NMR data of **5** there is lack of one signal from a proton bonded to a hydroxylated carbon. In the ^13^C NMR spectrum there is only one signal from the hydroxylated carbon atom (C-3 at δ 70.44 ppm) but the shifted signal appears at δ 201.20 ppm. The position of the signal corresponds to a six-membered ring. Signals from a double bond (δ_C_ 126.06 and 166.27 ppm; δ_H_ 5.74 ppm) and a hydroxyl group at C-3 (δ_C_ 70.44 ppm and δ_H_ 3.68 ppm) are visible evidence of the unchanged 3β-hydroxyandrost-5-en skeleton. This indicated oxidation of the hydroxyl group at C-7. The spectral data of the obtained compound **5** are consistent with the literature for 3β-hydroxyandrost-5-en-7,17-dione^61^. The NMR data for the compound **6** show a similar feature to that of compound **5**. Signals of carbons 3, 5, 6, 7 and 17 remain nearly unchanged, relative to **5**, indicating no change nearby these carbon atoms. However, in the ^13^C NMR spectrum, a signal at δ_C_ 68.37 ppm indicating a hydroxyl group in this compound was observed. This resonance is bound to a proton at δ 4.17 ppm (triplet of doublets). The COSY spectrum shows the coupling between this resonance and 9-H, 12-Hα and 12-Hβ. The only position that meets these requirements is C-11. Additionally, the new resonance does not couple with 18-CH_3_ and 19-CH_3_, which are oriented in the β position. The analogue **6** was deduced to be 3β,11α-dihydroxyandrost-5-en-7,17-dione. The NMR data for the compound **10** show no signal from the C-5 double bond either in ^1^H NMR (about 5.7 ppm) or in ^13^C NMR (130–170 ppm). These signals are moved to δ_H_ 2.01 (6-Hα), 2.89 (6-Hβ), δ_C_ 43.21 (C-5) and 45.39 (C-6). Movement of the C-7 signal at ^13^C NMR from 201.20 (characteristic for an α,β-unsaturated ketone such as **5**) to 211.13 ppm was observed. There are no additional signals. The rest of the proton and carbon signals are quite similar to **5**. Taking into account the above data, it follows that **10** is 3β-hydroxyandrostan-7,17-dione. Comparing **11** to **6** in the ^13^C NMR spectrum, there is a lack of to signals at δ 166.02 and 125.41 ppm, which refer to the double bond between C-5 and C-6. The characteristic signals C-11 (δ 68.24) and C-17 (217,32 ppm) remain nearly unchanged. The signal from C-7 was identified at 209.66 ppm. The signal from C-3 was shifted from 70.77 ppm to 210.89 ppm. This movement is a consequence of the oxidation of the hydroxyl group at the C-3 of **6** to the carbonyl moiety of **11**. In the ^1^H NMR spectrum, positions of 18-CH_3_, 19-CH_3_ and 11-Hβ signals and the shape of 11-Hβ definitely confirm the structure of compound **11** as 11α-hydroxyandrost-3,7,17-trione. The ^13^C NMR data for compound **7** show the appearance of three carbon atoms bonded to hydroxyl groups (δ 64.11, 68.86, 71.46 ppm), one carbon with a ketone moiety (δ 219.19 ppm) and two carbon atoms forming a double bond (δ 123.38, 147.32 ppm). Taking into consideration the intermediate product (**2**) and the HSQC data, we can assume that the carbon C-7 at δ 64.11 ppm is bonded to the proton at δ 3.97 ppm (ddd) and the carbon C-3 at δ 71.46 ppm to the proton at δ 3.59 ppm (tt). The carbon signal observed at δ 68.86 ppm is coupled to the proton at δ 4.12 ppm (td). The COSY data showed couplings between the proton at δ 4.12 ppm and 12-Hβ (δ 2.15 ppm), 12-Hα (δ 1.30 ppm) and 9-H (δ 1.37 ppm). Taken together, these data give 3β,7α,11α-trihydroxyandrost-5-en-17-one. The ^13^C NMR data for compound **3** show the appearance of a new carbon signal at δ 68.86 ppm which is linked with a proton at δ 4.12 ppm, compared to the DHEA (**1**) spectrum. The position of this signal in the NMR spectrum indicated the presence of another hydroxyl group. The chemical shifts of characteristic methyl groups (18-CH_3_ and 19-CH_3_) and the shape of the signal at 4.12 ppm (td) correspond to a proton in β orientation connected to C-11. This location is confirmed by inspection of the COSY data, which showed couplings between H-11β and both protons at C-12 and H-9. These three protons, in HMBC spectra, couple with the C-11 carbon. In the same spectra, the signal from the 11β proton is coupled with protons at C-9, C-10 and C-12. All the chemical shifts in ^1^H NMR and ^13^C NMR are compatible with those found in the literature for 3β,11α-dihydroxyandrost-5-en-17-one^46^. Compared to compound **3** from which it was derived, the ^13^C NMR data for **9** show the appearance of signals, at δ 169,90 ppm and at 81.71 ppm, which are not attributable to any proton. The presence of these carbon atoms implies the lactone moiety in compound **9**. Further inspection of NMR data revealed that the structure of the A-B ring of 3β-hydroxy-5-ene steroids remained unchanged, which suggests the lactonisation of a D ring. The investigated compound is 3β,11α-dihydroxy-17a-oxa-D-homo-androst-5-en-17-one, and its spectral data are in accordance with the literature^46^. The ^13^C NMR data for compound **8** show the appearance of signals, in comparison to compound **5**, at δ 171.73 ppm and at 82.43 ppm, which are characteristic for D-lactone^21^. Further inspection of NMR data revealed that the structure of the A-B ring of compound **5** remained unchanged, which confirms the lactonisation of a D ring. The investigated compound **8** is 3β-hydroxy-17a-oxa-D-homo-androst-5-en-7,17-dione and the spectral data are not described in the literature.

### Biotransformation of DHEA

To confirm the diversity of DHEA metabolism in the cultures of different strains of the species *Beauveria bassiana*, the tested substrate was transformed by characterised new isolates from this species. Additionally, DHEA metabolism was tested in the cultures of two new strains from the same genus, *Beauveria caledonica*. In order to investigate the metabolic pathway of DHEA by these biocatalysts, the composition of crude mixtures after various transformation periods was studied. Accumulations of products during transformation in cultures of different strains are compiled in Table 3.

**Table 3:** Product accumulation during the conversion of DHEA.

Microorganism	Compounds found in the reaction mixture [%]	Retention time by GC [min]	Biotransformation time [hours]							
			3	6	9	12	24	72	168	216
*Beauveria bassiana* KCh J1.5	(**1**)	4.87	99	21	—	—	—	—	—	—
	(**3**)	6.83	—	2	9	10	12	10	8	—
	(**2**)	6.89	—	70	80	77	49	13	1	—
*Beauveria bassiana* KCh J2.5	(**1**)	4.87	99	99	99	66	3	—	—	—
	(**2**)	6.89	—	—	1	7	24	27	27	26
	(**4**)	6.92	—	—	3	18	61	58	57	57
*Beauveria bassiana* KCh J1	(**1**)	4.87	99	86	68	18	—	—	—	—
	(**2**)	6.89	—	7	29	70	76	52	—	—
	(**5**)	7.53	—	4	3	2	3	6	—	—
	(**7**)	8.12	—	—	—	—	3	12	—	—
	(**6**)	9.51	—	—	—	—	7	21	—	—
*Beauveria bassiana* KCh J3.2	(**1**)	4.87	82	2	—	—	—	—	—	—
	(**2**)	6.89	10	54	53	51	48	45	18	15
	(**3**)	6.83	5	29	30	28	29	28	27	21
	(**5**)	7.53	—	2	2	4	5	6	12	27
	(**7**)	8.12	—	7	8	6	5	5	7	8
	(**6**)	9.51	—	3	3	4	4	6	10	12
*Beauveria bassiana* KCh BBT	(**1**)	4.87	97	49	—	—	—	—	—	—
	(**3**)	6.83	2	30	54	59	15	—	—	—
	(**2**)	6.89	1	9	19	11	9	—	—	—
	(**5**)	7.53	—	1	2	2	5	1	—	—
	(**6**)	9.51	—	—	3	3	5	9	9	9
	(**8**)	10.72	—	—	4	4	5	11	11	11
	(**9**)	11.18	—	5	15	20	53	66	65	64
*Beauveria caledonica* KCh J3.3	(**1**)	4.87	99	89	61	21	—	—	—	—
	(**2**)	6.89	—	5	18	36	28	6	—	—
	(**4**)	6.95	—	5	19	38	26	4	—	—
	(**5**)	7.53	—	—	1	3	11	6	—	—
	(**6**)	9.51	—	—	—	—	16	55	59	—
*Beauveria caledonica* KCh J3.4	(**1**)	4.87	85	8	4	1	—	—	—	—
	(**10**)	6.38	—	—	—	—	3	7	11	11
	(**2**)	6.89	5	29	30	29	34	24	10	—
	(**4**)	6.95	7	51	40	32	19	7	5	—
	(**5**)	7.53	—	6	15	21	16	9	7	—
	(**11**)	8.10	—	—	—	—	1	5	11	14
	(**6**)	9.51	—	—	4	8	19	44	58	58

Data are the average of 3 independent experiments. Standard errors were in the range: 0–5.

**Figure 1:**
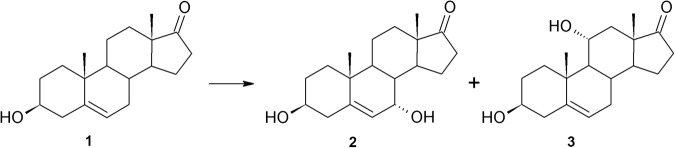
DHEA transformation in *Beauveria bassiana* KCh J1.5 culture.

Transformation of DHEA (**1**) in the five cultures of *Beauveria bassiana* strains was varied. In *Beauveria bassiana* KCh J1.5 culture, DHEA (**1**) was transformed to monohydroxylated products at 7α (**2**) and 11α positions (**3**) (Fig. 1). The major product was **2**. The whole added substrate (**1**) was transformed in less than 9 hours, but after 24 hours of conducting the process products were degraded into many compounds (9–11) in a small amount (1–14%). To the authors’ best knowledge, all available papers describe 11α-hydroxylation of DHEA or its C-17 reduced analogue (androstenediol)^46,51,57^, but only Huszcza *et al*.^24^ describe hydroxylation in the 7α position in the *Beauveria bassiana* culture. In contrast to the cited paper, in this study the 7α-product was in the majority.

**Figure 2:**
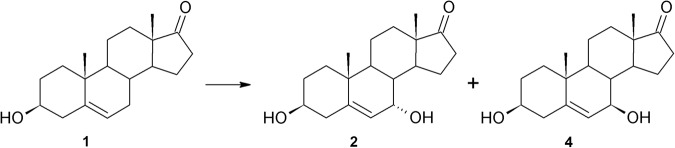
Hydroxylation of DHEA in *Beauveria bassiana* KCh J2.5 culture.

Also, *B*. *bassiana* KCh J2.5 transformed DHEA into two monohydroxylated products, but both at position 7: **4** and **2**, where **4** was in the majority (Fig. 2 and Table 3). Substrate conversion in the KCh J2.5 strain took place within 24 hours. Degradation of products was not observed. Placement of the hydroxy group at the 7β position in transformation by *B*. *bassiana* has not been described so far. However, in the literature, there is plenty of evidence of hydroxylation, selective or nonselective, at C-7 of the steroid skeleton by microorganisms with various yields^22,62–64^. The formation of both C-7 epimers, as well as **3**, may be the result of one enzyme’s action, as it was discussed by Milecka-Tronina, relative to 7α, 7β, 9α and 11α- hydroxysteroids^65^. Distances between oxygen atoms at C-3 or C-17 and hydroxylated hydrogen atoms are similar, so there is a possibility of normal, reverse inverted or inverted binding between substrate and enzyme. The hypothesis of the action of a single hydroxylase cannot be, however, established for certain on the basis of the obtained results. The relevance of 7-hydroxy derivatives was discussed at the beginning of the paper.

**Figure 3:**
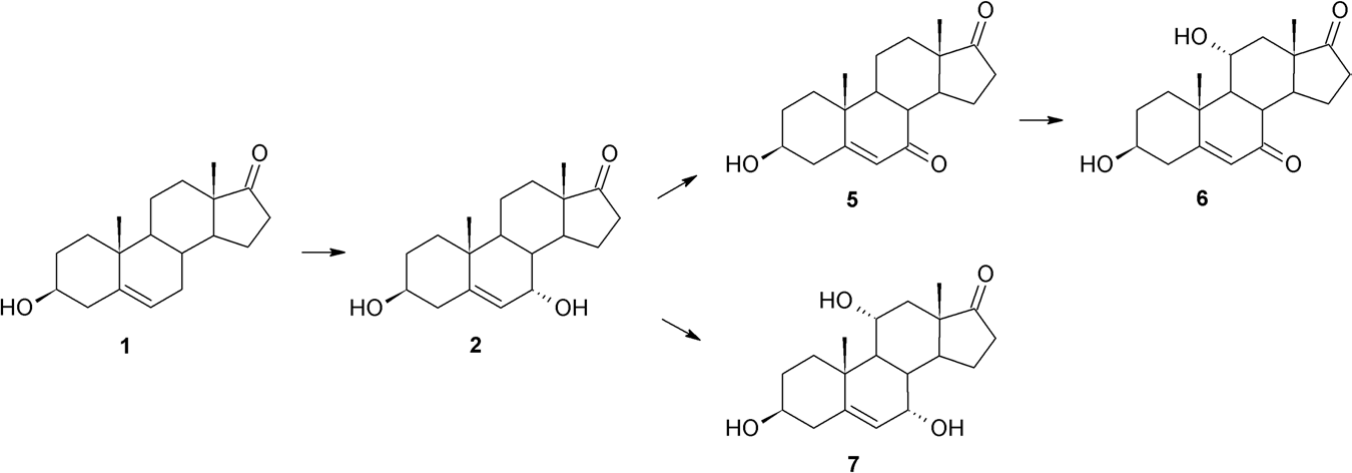
Transformation of DHEA in *Beauveria bassiana* KCh J1 culture.

In the *B*. *bassiana* KCh J1 culture substrate (**1**) was stereoselectively hydroxylated to **2**, then oxidised to **5** and then again hydroxylated but in the 11α position to **6** (Fig. 3). Simultaneously, **2** underwent hydroxylation in the 11α position, forming **7**. Formation of **5**, **6** and **7** in *Beauveria bassiana* culture was observed for the first time. Fast transformation of DHEA in the culture of this strain may be a source of **2** due to about 70% conversion in 24 hours. After 72 hours of the process, all products were degrading, and on day 7 of the process none of them was observed (Table 3).

**Figure 4:**
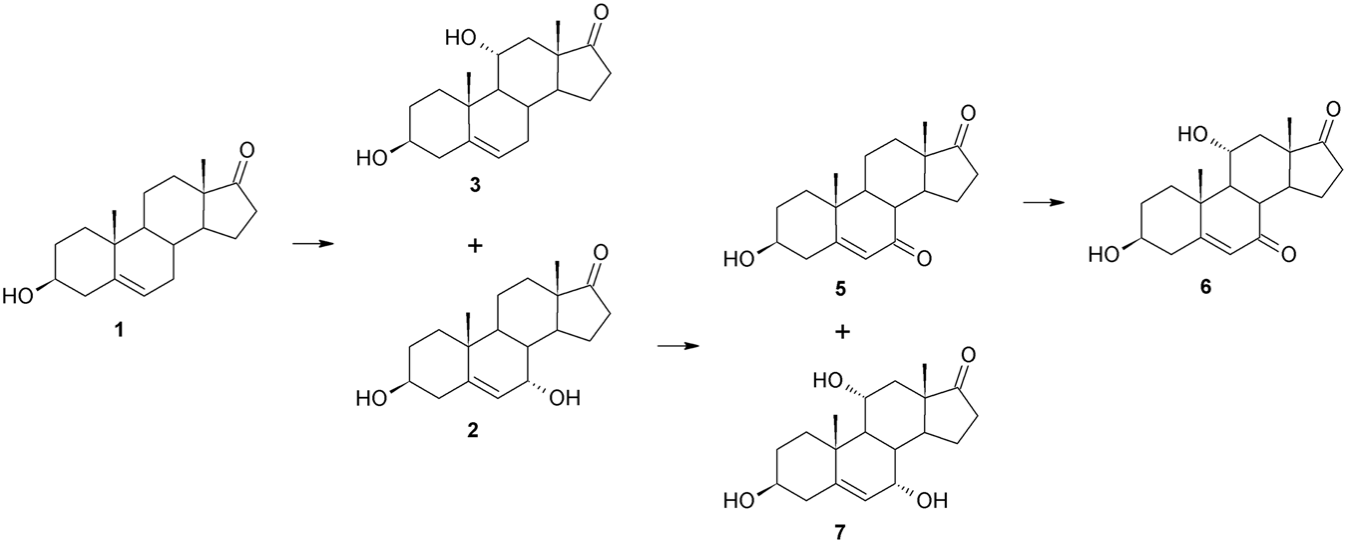
Cascade reactions of DHEA in *Beauveria bassiana* KCh J3.2 culture.

Comparable transformation of DHEA (**1**) was carried out by *B*. *bassiana* KCh J3.2 (Fig. 4). Apart from all the transformations taking place in the KCh J1 culture, hydroxylation at the 11α position of DHEA occurs additionally. Compound **3** was not transformed further, e.g. to **9** as it was in the KCh BBT culture. As in the KCh J1 culture, **2** occurred in a higher concentration than **3** (Table 3). Compared to KCh J1.5 transformation, in the *B*. *bassiana* KCh J3.2 culture all added DHEA was transformed faster, in six hours, and the products were not degraded.

**Figure 5:**
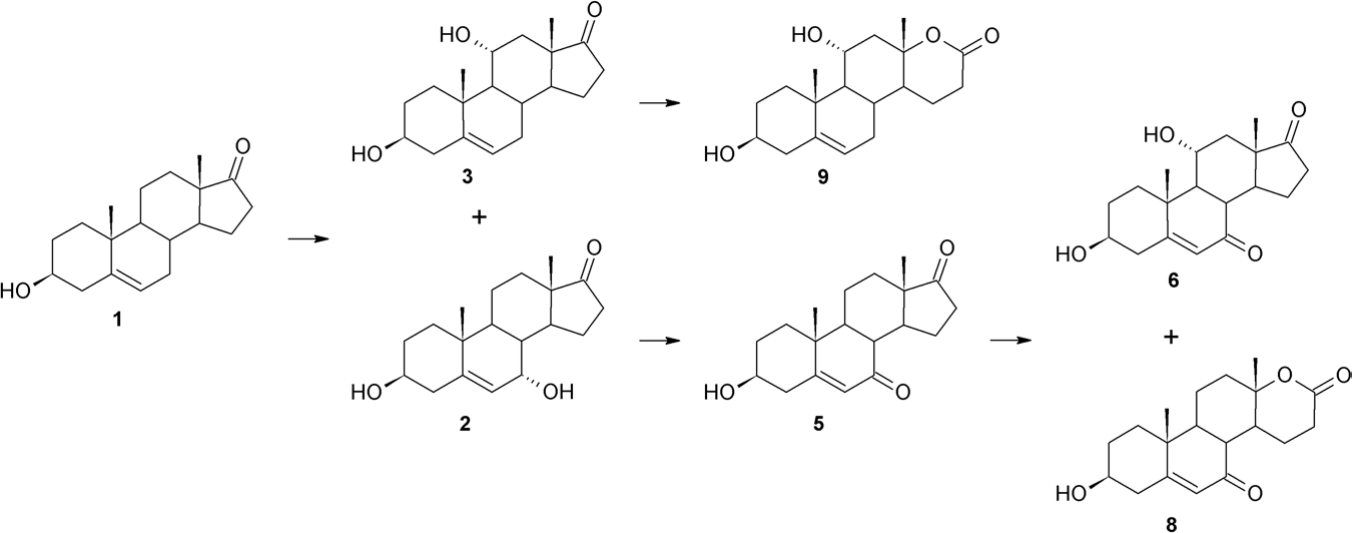
Transformations of DHEA in *Beauveria bassiana* KCh BBT culture.

In *Beauveria bassiana* KCh BBT at the beginning of the process there occur similar hydroxylations to other tested species – at the 7α or 11α position (Fig. 5) – but further conversions are slightly different. The strain provides stereoselective hydroxylation of the C-7 carbon atom in position α, then oxidation of this group and then another hydroxylation in the 11α position, giving **2**, **5** and **6**, respectively. The described pathway is mutual to the KCH J1 and KCh J3.2 strains. Furthermore, **5** underwent lactonisation in the D-ring to give **8**, not described in the literature. Products of this pathway formed a minority of the reaction mixture. The majority were products of hydroxylation at the 11α position – **3** – which is a step to lactonisation of a D-ring – **9**. A similar relationship between the concentration of 7α (**2**) and 11α-hydroxy-DHEA (**3**) was observed by Huszcza *at al* in the cultures of *B*. *bassiana* AM446^24^ but in *B*. *bassiana* KCh J1.5 and KCh J3.2 inverted. *Beauveria bassiana* KCh BBT strain transformed all added DHEA in less than 6 hours. The highest concentration of **9** was observed after 3 days of conducting the process, reaching 66% (conversion by GC), and it remained stable until the end of the process. In contrast to *Beauveria bassiana* ATCC 7159^51^ and AM446^24^, none of the strains tested in this study reduced the C-17 carbonyl group before hydroxylation at the 11α position. The ability to form steroid D-lactones is characteristic mainly for strains from the genera *Penicillium* and *Aspergillus*^66–69^, but only *Beauveria bassiana* and *Isaria fumosorosea* have the capacity for hydroxylation and then Baeyer-Villiger oxidation of the steroid skeleton^27,44,46,70^.

**Figure 6:**
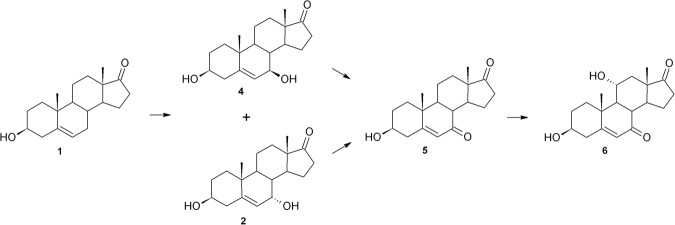
Transformations of DHEA in *Beauveria caledonica* KCh J3.3 culture.

**Figure 7:**
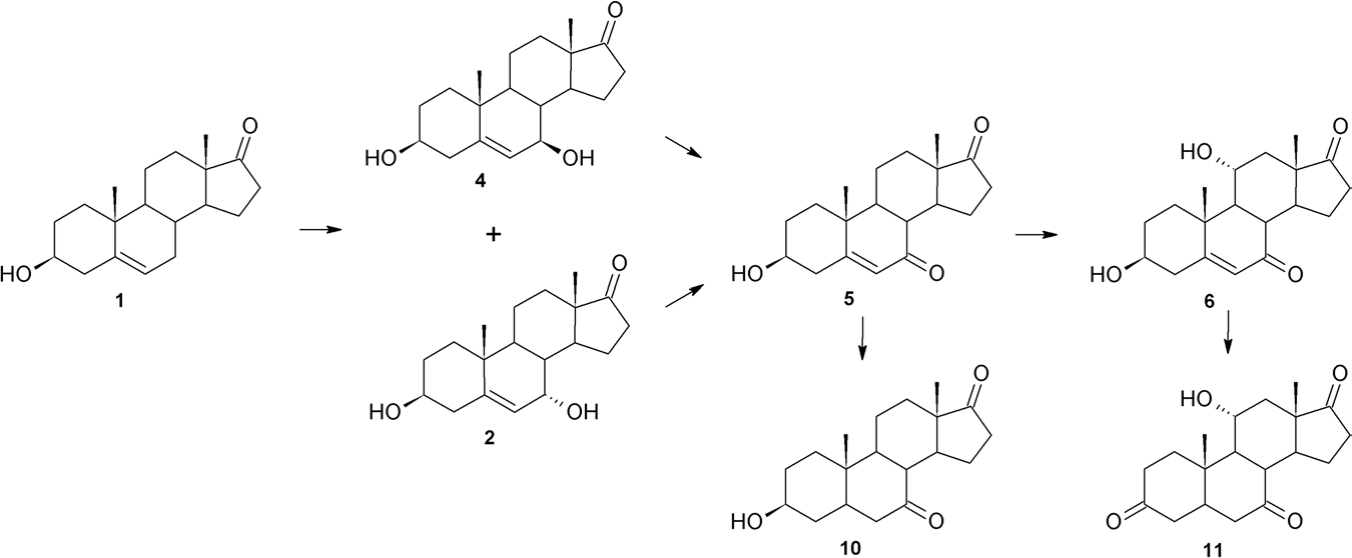
DHEA biotransformation in *Beauveria caledonica* KCh J3.4 culture.

Transformation of a steroid compound such as DHEA in *Beauveria caledonica* is described here for the first time. In contrast to *B*. *bassiana*, transformation of DHEA in both tested strains of *B*. *caledonica* is similar. The first stage of the process provided by the strains of *Beauveria caledonica* KCh J3.4 and KCh J3.3 was hydroxylation of DHEA (**1**) in the 7-C position, which gave two products: **2** and **4** (Figs 6 and 7). Then, the hydroxyl groups of both structures were oxidised to **5**. The resulting product was hydroxylated at the 11α position, leading to **6**. Additionally, in the culture of *B*. *caledonica* KCh J3.4, the reduction of double bonds of **5** (leading to **10**) and **6** was observed. Moreover, the hydroxyl group in the C-3 position of **6** was oxidised, giving finally **11**.

Differences in activity and metabolic ability between two strains of the same species are indicated. The *B*. *caledonica* KCh J3.3 strain transforms DHEA (**1**) more slowly than KCh J3.4. The whole amount of added substrate was metabolized in 12 or 24 hours in the *Beauveria caledonica* KCh J3.4 or *B*. *caledonica* KCh J3.3 culture, respectively. Moreover, in the broth of *Beauveria caledonica* KCh J3.4 **4** occurred faster and in a higher concentration than its epimer (51% of **4** vs 29% of **2** at the 6^th^ hour of the process) (Table 3). In the *B*. *caledonica* KCh J3.3 culture both epimers appeared in nearly equal concentrations, maximally 37% of **2** and 40% of **4** at 24 h after addition of the substrate. Additionally, final metabolites of **1** were not degraded in *B*. *caledonica* KCh J3.3 culture in 9-day transformation. Furthermore, only one of the strains (KCh J3.3) has the ability to reduce the C-5 double bond.

## Conclusions

The aim of this study was to evaluate the differences in metabolism of the same substrate in the cultures of five different strains of the same species – *Beauveria bassiana*. Additionally, the transformation of DHEA (**1**) by two strains of *Beauveria caledonica* was tested. All strains used in this study were isolated from arthropod cadavers and then molecularly identified using analysis of the ITS1-ITS2 rDNA sequence.

All tested *Beauveria bassiana* strains transformed the substrate with high conversion (100% in 12 h). The 7α and 11α-hydroxy derivatives described in the literature were observed as well as lactonisation of a D-ring. We also described new DHEA metabolic pathways which gave two products not described before: 3β-hydroxy-17a-oxa-D-homo-androst-5-en-7,17-dione (**8**) and 3β,11α-dihydroxyandrost-5-en-7,17-dione (**6**). The cascade of reactions observed in all tested strains was varied.

Transformation of the steroid substrate by *Beauveria caledonica* was described for the first time. DHEA was hydroxylated at 7α, 7β and 11α positions. Oxidation reactions of the hydroxy group at C-7 and reduction of the double bond were also observed.

All tested strains from *Beauveria* genera effectively transformed the steroid substrate using several different enzymes, resulting in cascade transformations and new products.
